# Type I Collagen-Fibrin Mixed Hydrogels: Preparation, Properties and Biomedical Applications

**DOI:** 10.3390/gels6040036

**Published:** 2020-10-20

**Authors:** Thibaud Coradin, Kun Wang, Thalie Law, Léa Trichet

**Affiliations:** Sorbonne Université, CNRS, Laboratoire de Chimie de la Matière Condensée de Paris, 4 Place Jussieu, 75005 Paris, France; kun.wang@sorbonne-universite.fr (K.W.); thalielaw@gmail.com (T.L.); lea.trichet@sorbonne-universite.fr (L.T.)

**Keywords:** type I collagen, fibrin, hydrogels

## Abstract

Type I collagen and fibrin are two essential proteins in tissue regeneration and have been widely used for the design of biomaterials. While they both form hydrogels via fibrillogenesis, they have distinct biochemical features, structural properties and biological functions which make their combination of high interest. A number of protocols to obtain such mixed gels have been described in the literature that differ in the sequence of mixing/addition of the various reagents. Experimental and modelling studies have suggested that such co-gels consist of an interpenetrated structure where the two proteins networks have local interactions only. Evidences have been accumulated that immobilized cells respond not only to the overall structure of the co-gels but can also exhibit responses specific to each of the proteins. Among the many biomedical applications of such type I collagen-fibrin mixed gels, those requiring the co-culture of two cell types with distinct affinity for these proteins, such as vascularization of tissue engineering constructs, appear particularly promising.

## 1. Introduction

Protein-based hydrogels are of paramount importance in the biomedical field [1,2,3,4,5]. They combine biocompatibility, biodegradability and high hydration with the ability to present specific amino acid sequences that can be recognized by cells to favor their adhesion, proliferation and differentiation [6,7,8,9,10]. Of particular interest are those prepared from fibrillar proteins [11]. In vivo, many of these proteins, such as several collagens, silk fibroin or keratin can form complex hierarchical structures constituting the architecture of most tissues [12,13,14]. However, mimicking such structures in vitro is highly challenging [15,16,17,18], as they are the results of multiple cellular events involving the synthesis and processing of many biomolecules as well as a variety of biophysical cues

Nevertheless, even simple hydrogels prepared from a single protein can recapitulate enough features of extracellular matrices, such as biochemical composition, structure or mechanical properties, to be used as biomaterials [19,20]. Varying physico-chemical parameters such as concentration, pH or salinity offers a wide variety of structures with tailored organization and mechanical properties [21,22,23,24]. Many processing methods, including most recent 3D printing, provide further tools to adapt the hydrogel features to a specific application [25,26,27,28]. Physical, chemical or enzymatic cross-linking [29,30] as well as incorporation of particles, including calcium phosphates, silica or carbon nanotubes, following a composite approach can also be undertaken to improve the mechanical properties of the hydrogels as well to increase their stability in water and slow down their biodegradation [31,32,33].

However, to increase the ability of the hydrogels to control the fate of associated cells, for instance for the guidance of axon growth from neural cells or to orient the differentiation of mesenchymal stem cells, it can be interesting to incorporate additional biological moieties to the protein-based network [16,34,35]. One option would be to graft specific peptides on the proteins, as previously performed for synthetic self-assembling systems [36], but this may impact on the course of the fibril formation process (fibrillogenesis). Another recently-described option is incorporate particles that are surface-decorated with these peptides [37]. However, a more straightforward approach is to prepare mixed hydrogels combining two biological polymers with complimentary biological functions, such as type I collagen-hyaluronic acid constructs for wound healing and tissue engineering [38,39,40].

In this review, we illustrate this approach by examining how two fibrillar proteins, type I collagen and fibrin, can be associated within mixed hydrogels. After a short presentation of these two proteins that will emphasize their similarity and difference, we will describe the various strategies that have been developed to associate them within a hydrogel. In particular, methodologies allowing for the co-fibrillogenesis of the proteins will be highlighted. Insights into the structure of these gels, especially the connections between the type I collagen and fibrin networks, will be provided, together with their mechanical properties. Finally, the reported biomedical applications of such mixed constructs will be presented, with specific emphasis on the cardiovascular area.

## 2. A Short Presentation of Type I Collagen and Fibrin

### 2.1. Type I Collagen

**Figure 1:**
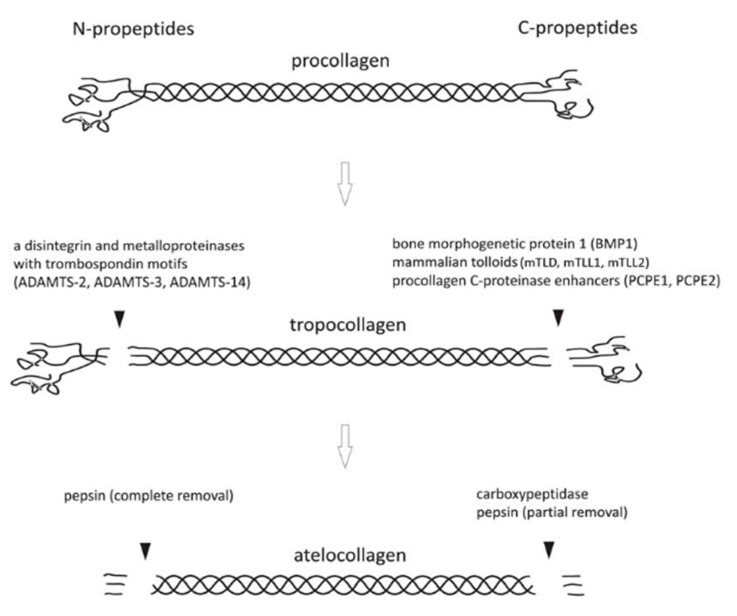
Type I collagen is biosynthesized as *procollagen*; enzymatic cleavage of terminal propeptides forms *tropocollagen*; further non-specific enzymatic cleavage of terminal telopeptides yields to *atelocollagen*. Reproduced with permission from [47]. Copyright The Royal Society of Chemistry 2012.

Collagens form a family of proteins that constitute more than one-third by weight of body protein tissue and are the most prevalent components of extracellular matrices (ECM) [41]. Collagens consist of three polypeptide strands held together in a helical conformation with about 28 different types described, with types I, II, III, and IV being the most common [42,43]. These parallel-arranged strands are entangled in a left-handed polyproline II-type (PPII) helix and wrap around each other to form a right-handed triple helix, which is stabilized by interchain hydrogen bonding and intrachain *n*→π* interactions [44]. The intracellular form of type I collagen, termed *procollagen*, includes N- and C-terminal peptides that allow it to be soluble in the cytoplasm conditions (Figure 1).

Upon externalization, these peptides are cleaved forming *tropocollagen* molecules with a MW of 300 kDa, exhibiting a rod-like shape 1.5 nm in diameter and 300 nm in length [45]. These molecules can self-assemble by a quarter-stagger package pattern to form proto- and micro-fibrils subunits ultimately leading to fibrils up to several microns in length. Fibrils can further assemble as fibers at higher scales. Intrafibrillar cross-linking occurs by the enzymatic action of lysyl oxidase, between lysine or hydroxylysine residues and the specific active binding sites present in neighboring triple helices [46]. In vitro, N- and C-terminal telopeptides can also be enzymatically cleaved, resulting in *atelocollagen* [47]. In this form, collagen is soluble at pH 7 and has a lower immunogenicity than tropocollagen but cannot form fibrils anymore.

Type I tropocollagen molecules are easily extracted from fresh tissues, especially from young rat tails tendons, and purified, allowing for their extensive physico-chemical characterization. One of their remarkable features is that they can be considered as anisotropic rigid charged colloids over a wide range of pHs. This allows to prepare homogeneously-dispersed solutions at very high concentration, up to 300 mg mL^−1^ near pH 3. Over this range different regimes of interactions were identified: dilute (<2.5 mg mL^−1^), semi-dilute (ca. 2.5–15 mg mL^−1^), repulsive (ca. 15–100 mg mL^−1^) and attractive (>100 mg mL^−1^), the boundary concentrations depending on the exact pH and ionic strength [48]. Solutions where repulsive interactions are prevailing are sometimes termed “concentrated” while those with higher collagen content can be referred as “dense”. The distinction is of tremendous importance because the existence of an attractive regime is correlated with the formation of liquid crystal phases [49]. Such organizations do exist in natural tissues, such as cornea or bone, and can be reproduced in vitro, at least to some extent [50].

The in vitro synthesis of type I collagen networks from tropocollagen (usually simply designated as collagen) is known for many years and has been largely reviewed (see for instance [51,52,53]). The basic principle relies on the use of a type I collagen solution in acidic medium which pH is increased to neutral. Since the isoelectric point (*iep*) of collagen is ca. 8.2, increasing the pH decreases the positive charge of the protein chains until repulsive electrostatic interactions become weak enough to allow their stacking and the formation of fibrils. Noticeably, neutralization can be performed by addition of buffer or sodium hydroxide for diluted solutions thanks to their low viscosity. However, for concentration larger than ca. 5 mg mL^−1^, the high viscosity and fast gelation requires the use of another approach, i.e., a vapor phase process using ammonia [54]. This has a very practical consequence, as the liquid phase process allows for incorporation of cell suspension and therefore 3D encapsulation within the collagen network [55], whereas the vapor phase is not compatible with direct immobilization. One smart solution to address this limitation was described by Brown et al. some years ago based on cell immobilization within diluted collagen hydrogels followed by their densification under compression [56,57].

Besides concentration, many other physico-chemical conditions (pH, temperature, ionic strength, chemical nature of electrolytes) and additives impact the fibrillogenesis process and the structure of the resulting hydrogels, as recently extensively reviewed by Zhu et al. [53]. For example, collagen fibril diameters can vary from ca. 20 nm to 1 μm as a function of concentration [48]. Fiber length can vary from 2 μm to 50 μm as a function of ionic strength and from 2 μm to 30 μm as a function of temperature [58]. Note that such variations also impact the porosity of the hydrogels. Storage moduli of hydrogels are below 1 kPa in the dilute regime and reach 10 kPa at ca. 40 mg mL^−1^ [59]. Young moduli of dense hydrogels can be of several GPa [60].

Because it is the main component of most human tissues, type I collagen has found applications in almost all fields of biomaterial science, from research to the clinic [53,61,62,63,64,65]. However, commercial products are rarely provided in a hydrogel (i.e., hydrated) form but rather in a dried form. Moreover, they are usually chemically or enzymatically-cross-linked, for instance in widely-used collagen sponges or membranes, with the main goal of slowing down their biodegradation [64]. In the case of bone repair, the collagen matrix is in most cases associated with calcium phosphate particles [65]. In the research area, type I collagen hydrogels are studied over a broad range of conditions, including mixing with other natural or synthetic polymers or incorporation of nanoparticles for drug release applications [53,59,60,61,62,63]. Recent trends include new materials processing approaches [66] and use of collagen scaffolds as hosts for stem cells both in vitro and in vivo [67].

### 2.2. Fibrinogen and Fibrin

**Figure 2:**
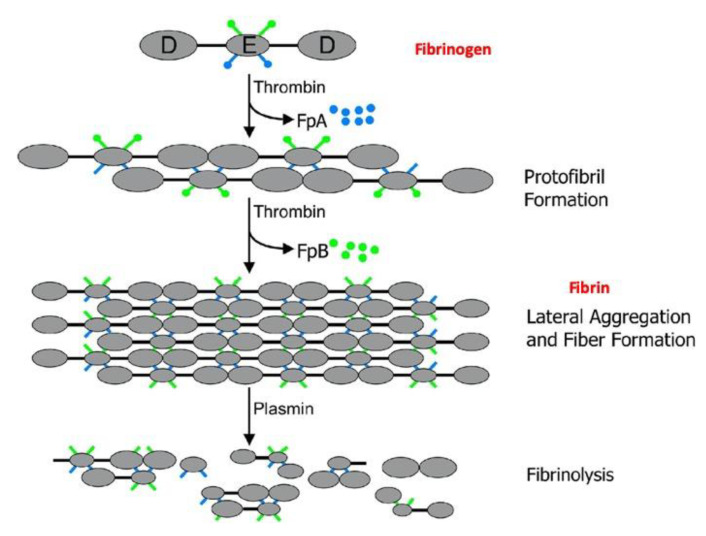
Fibrinogen is converted into fibrin via a two-step enzymatic reaction by thrombin. The cleavage of fibrinopeptide A (FpA) allows for protofibril formation. The cleavage of fibrinopeptide B (FpB) allows for later aggregation leading to fibrin fibril formation. In vivo, the plasmin protease is able to degrade the fibrin fibers. Adapted and reproduced with permission from [69]. Copyright 2006 Elsevier Ltd.

After blood vessel injury, platelets flowing in bloodstream respond by adhering to exposed subendothelial matrices of the vessel walls [68]. This triggers platelet activation and aggregation, forming a primary plug. Subsequently, circulating prothrombin undergoes a series of enzymatic reactions which leads to the production of thrombin as a smaller protein. The resulting thrombin can then interact with fibrinogen to form a fibrin mesh to reinforce the plug [69]. Fibrinogen molecules have a MW of 340 kDa and their shape can be described as elongated 46 nm-long structures with a section of ca. 5 nm [70]. This structure consists of two outer D regions, each connected by a coiled-coil segment to the central E region, and the protein is made up of three pairs of polypeptide chains, (AαBβγ)_2_, held together by 29 disulphide bonds [71,72] (Figure 2). The N-terminal part of all six chains are joined in the central region, that is connected to the end domains by α-helical coiled coils. The central region also contains the two pairs of fibrinopeptides. Thrombin, generated by the effect of platelet on prothrombin, cleaves fibrinopeptides from the N-terminal parts of the Aα and Bβ chains, FpA and FpB, respectively, the cleavage of FpB being delayed compared to that of FpA [73]. Cleavage of the A fibrinopeptides exposes knobs ‘A’ that are complementary to pockets or holes ‘a’ which are located in the γC-nodules of another fibrin molecule. There are also knobs ‘B’ exposed by the removal of the B fibrinopeptides that are complementary to holes ‘b’ in the βC-nodule. Binding of the central region of one molecule to the end of an adjacent molecule by the specific, quite strong and stable interactions between knobs ‘A’ and holes ‘a’ yields fibrin oligomer in which the fibrin monomers are half-staggered (Figure 2) [74]. B fibrinopeptide cleavage occurs primarily from fibrin oligomers and enhances lateral aggregation. The γ- and β-nodules of fibrinogen contain high- and low-affinity calcium-binding sites. High-affinity calcium-binding residues in the γ-nodule are critical for protofibril formation while lower affinity ones contribute to the extent of lateral aggregation [72]. Some studies showed that calcium ions can accelerate the conversion of fibrinogen to fibrin by thrombin, and that clotting times–i.e., time required to form a fibrin network after thrombin addition, and also termed lag time- were shortened with increasing concentration of Ca^2+^ [75]. Blood coagulation factor FXIII is essential for maintaining hemostasis by stabilizing the fibrin clot and protecting fibrin against fibrinolysis independently of other plasma proteins [76]. Such a stabilization occurs through the formation of ε-(β-glutamyl)lysyl covalent bonds between γ-γ, γ-α and α-α chains of adjacent fibrin molecules.

Many physico-chemical factors including concentration, ionic strength, pH, and temperature can influence the formation of fibrin network from fibrinogen [21,77,78,79,80]. Importantly these parameters can influence both the structure of the fibrinogen molecules and the fibrillogenesis reaction. Some representative results are presented hereafter, obtained from in vitro studies performed in simple conditions, usually using fibrinogen, thrombin and calcium mixtures. Although the resulting systems lack the complexity of in vivo clots and may more suitably be termed fibrin hydrogels, the two terms are sometimes mixed in the literature. Concentration of fibrinogen in human plasma is ca. 3 mg mL^−1^. Fibrin hydrogels are commonly prepared from fibrinogen solutions in the 1–40 mg mL^−1^ range. Beyond this value, it is practically difficult to ensure a homogeneous dispersion of the proteins. Moreover, in common thrombin concentration conditions, the lag time becomes very short (<1 min). Typical storage modulus for fibrin hydrogels is below 1 kPa below 5 mg mL^−1^ fibrinogen concentration and reaches 10 kPa for 30 mg mL^−1^ fibrinogen concentration [81]. Elongation at break can be >200% [82]. It has to be pointed out that all these properties are strongly dependent on the thrombin concentration as higher content in this enzyme leads to thinner fibrin fibers, which can be 100 times stiffer than thick ones [80]. Viscoelastic properties of the gels also depend on their structural features, which include fiber size, density and branching point density [83]. For example, the ultimate tensile stress of gels prepared at 4 mg mL^−1^ fibrinogen decreased from 40 kPa to 10 kPa when thrombin concentration increased from 0.001 UT mg^−1^ to 0.1 UT mg^−1^ [84].

The effect of pH variations from 5 to 9 on the secondary structure conformation of fibrinogen has been studied [85]. With the pH increase, α-helicity decreased and β- strands increased. In parallel, near its iep (ca. 5.8), fibrinogen adopts a rather compact structure whereas below pH 4 and above pH 8, at low ionic strength, it exhibits a more extended conformation. Below pH 5.5, fibrin gels cannot be formed but, even in acidic conditions, short and thin fibrinogen protofibrils could be observed [86]. In this context, it was also shown that fibrinogen alone can form gel in acidic conditions after incubation at 37 °C. Both pH and temperature contribute to the denaturation of fibrinogen molecules that could aggregate and form a non-fibrillar network [87]. Clotting time tends to increase with ionic strength but it was shown to decrease with increasing Na^+^ concentration, which was attributed to a modification of thrombin-fibrinogen interactions [88]. Ionic strength and pH also influence the properties of the hydrogels. For example, for an ionic strength I = 0.15 (close to physiological conditions), opaque hydrogels constituted of thick fibrin fibers are obtained below pH 8 while transparent ones are formed at higher pH. However, for I = 0.45, opaque gels are obtained up to pH 6.5 only. Finally, going down from 37 °C to 15 °C led to an increase in lag time and the rate of fibrillogenesis but final turbidity of the gel, indicative of fibrin fiber size, was increased. This indicates that temperature not only influences fibrin monomer generation but also fibrin assembly [89].

Fibrin gels present many advantages, such as excellent biocompatibility and tunable porosity providing sufficient surface area and space for cell adhesion, proliferation and extracellular matrix regeneration [80,90]. They are currently used in clinics as haemostatic sealants, as glues or dressings, and are widely studied for applications in tissue engineering and bioengineering [91,92,93,94,95,96,97,98]. Most of the research data are obtained using of purified fibrinogen. However another popular source, most frequently used for pre-clinical or clinal studies, is obtained from platelet concentrates, obtained after centrifugation of whole blood to recover and eliminate red blood cells [99]. Such concentrates can be prepared with various contents of proteins (including fibrinogen) and cells (platelets, leukocytes) [100]. Although the precise terminology varies in the literature, solutions enriched in cells are most usually named platelet-or leukocyte-rich plasma (P-PRP and L-PRP, respectively) solutions. These solutions are usually prepared in the presence of an anticoagulant so that thrombin has to be added (or produced in vivo) to obtain an artificial clot. Formulations containing high amounts of proteins are termed platelet- or leucocyte-rich fibrin (P-PRF or L-PRF, respectively). They are mostly prepared in the absence of anticoagulants, i.e., they already contain clots, although the preparation of liquid PRF was also reported [101]. The benefit of these sources is that platelets can produce a large range of bioactive proteins, including angiogenic and growth factors, that can favor cell activity and tissue remodeling [102]. Leucocytes-free formulations, such as thrombocyte-rich solution (TRS) appear more favorable to reduce immune reaction [103]. Meanwhile, it has been argued that clots formed from PRF concentrate were more likely to have an efficient therapeutic effect because they are formed in more physiological conditions [100].

Among recent examples of the use of fibrin hydrogels in tissue engineering, highly-concentrated (40 mg mL^−1^) fibrin hydrogels were used as sub-retinal implants. Small size hydrogels could directly injected through the sclera of pigs via a home-made device and were degraded over 8 weeks [104], allowing for the establishment of a new interface between retinal pigment epithelium and retina. In the field of cardiac repair, a bioreactor system with mechanical stimulation was developed allowing to obtain a new tissue resembling native muscle in terms of structure, gene expression and maturity. The myoblasts arrangement was consistent with the orientation of the scaffold into highly organized fibrin fibrils, which yields to more mature myotubes with better sarcomeric patterning, diameter and length. At same time, myogenic genes showed a faster expression compared to original fibrin gel [105]. In the field of bioactive molecules delivery, aptamer-modified fibrin hydrogels were prepared that allowed for the controlled delivery of VEGF, enhancing the repair of critical size cranial defects [106]. As a final example related to cell delivery, thrombin was modified with cationic and surfactant moieties to enhance its affinity with biological membranes while avoiding its internalization. This construct was made to interact with hMSC and then fibrinogen solution was added, leading to the formation of the fibrin hydrogels from the cell surface. This strategy was validated in a Zebra fish model [107].

### 2.3. Comparison

To conclude this first part, it is important to point out some common features and major differences between these two kinds of hydrogels that would impact not only the conditions of preparation of mixed systems but also allow to anticipate some of their structural or biological features [108]. A first point to emphasize is related to the fact that in the case of type I collagen, the usual form used for hydrogel formation is the tropocollagen molecule, i.e., a form that has already been converted from the inactive procollagen state by cleavage of some terminal peptides. In contrast, fibrin gels are prepared from the inactive fibrinogen molecules and thus require an additional activation step via central peptide cleavage by thrombin to allow their self-assembly. This has a major impact on processing conditions as collagen fibrillogenesis can be triggered by a simple pH change while fibrin gel formation requires the presence of an additional reagent.

**Figure 3:**
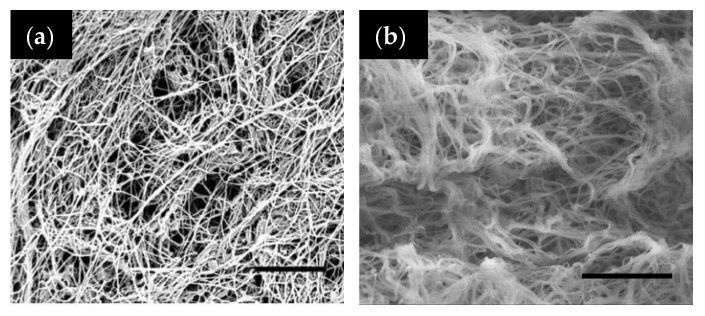
SEM images of (**a**) type I tropocollagen hydrogel at 5 mg mL^−1^ and (**b**) fibrin hydrogel at 6 mg mL^−1^. Scale bar: 5 μm for both images. (**a**) is reproduced with permission from [55]. Copyright 2010 John Wiley & Sons, Ltd.

From a structural perspective, tropocollagen and fibrinogen have close MW (300 kDa and 340 kDa, respectively) and have both a rod-like shape but the length and length-over-radius (aspect ratio) of the former (300 and 200 nm, respectively) are much higher than for the latter (ca. 45 nm and <10, respectively). A first consequence is that collagen fibrils originate from the assembly of single tropocollagen molecules, with typical periodic distance of 67 nm, whereas fibrin fibers are described as aggregates of protofibrils, with typical periodic pattern of 22.5 nm (i.e., half the length of a single molecule). Another important effect is that, at a fixed concentration, more fibrinogen than collagen molecules are required to form of a fiber of a given dimension, so that fibrin gels will be less dense (i.e., more porous) (Figure 3).

Considering interactions of cells with the two types of hydrogels, several reports show better adhesion on fibrin- than on collagen-based constructs, which may be attributed to stronger interactions of some integrins with the former [109,110,111]. In terms of the in vivo fate of these materials, one should point out that fibrin clots are only transitory materials that must be formed and degraded rapidly whereas collagen-based tissues must show long-term stability in the body. In humans, it has been shown that the turn-over rate of fibrinogen is ca. 3 days while that of collagen highly depends on the considered tissue but is ca. 15 years in skin [112,113]. Indeed, mechanisms for collagen biodegradation exist and two main enzymatic pathways are considered, one intracellular (cathepsins) and one pericellular (collagenases), originating from collagen-producing cells [114]. In contrast, fibrin is degraded by plasmin, an enzyme sustainably produced in an inactive circulating form, plasminogen, produced in the liver [115]. From a biomaterial perspective, it means that, in the absence of cross-linkers, fibrin-based hydrogels are usually biodegraded over a few days while collagen-based ones can be retained in vivo over weeks period [59,116]. This can explain, for some part, why the research related to collagen-based biomaterials is currently far more active than that dedicated to fibrin-based ones (a search on the topic ‘collagen biomaterials’ leads to >2000 articles over the last 5 years in the Web of Science database, whereas ‘fibrin biomaterials’ leads to <400).

## 3. Type I Collagen-Fibrin Mixed Hydrogels: Preparation

### 3.1. From Pre-Formed Collagen Materials

Two main strategies can be followed to associate type I collagen and fibrin. In the first one, a material containing only one of the proteins is prepared and then the second protein is introduced in a soluble form and its gelation is triggered. This first strategy has been mostly used to modify collagen-based materials, such as sponges or membranes, with fibrin, with the main goal of improving the integration of the biomaterial to the surrounding tissues [117,118]. The porous structure of the initial material and the impregnation method regulate the penetration depth of the fibrin precursor solution so that the fibrin gel may be formed only on the surface of the collagen scaffold, resulting in a bi-layered construct, or fill the whole porous volume [101]. One example reports collagen hydrogel modification by fibrin, by impregnation of the initial hydrogel by fibrinogen followed by contact with thrombin [119]. Most of the literature dedicated to such kind of materials reports on their biomedical properties, both in vitro and in vivo. However, data are scarce and poorly conclusive on the influence of the fibrin layer or network on the structural and mechanical properties of the collagen scaffold [120]. They will therefore not be discussed afterwards.

### 3.2. From Protein Mixtures

**Figure 4:**
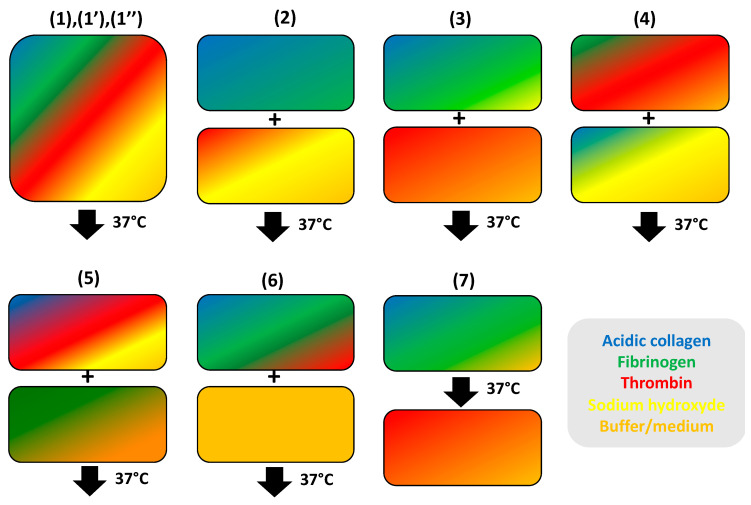
Schematic representation of various reported protocols for the preparation of mixed type I collagen-fibrin hydrogels from protein mixtures. Each component is represented by a color code, as indicated in the grey box. Note that in all protocols except (**7**), gel formation is always triggered by incubation at 37 °C after all components have been mixed.

Indeed, stronger interactions between the two proteins are expected to arise if they can be mixed in a pre-polymerized form before triggering their simultaneous, or at least conjunct, self-assembly. Several methods have been developed along this strategy. Key elements in these protocols are usually fibrinogen, thrombin and tropocollagen plus a base (usually NaOH) and/or a buffer (in cell-free experiments) or a culture medium to achieve neutral pH conditions (Figure 4).

A first standard protocol (1) developed by Stegemann et al. [121] is the sequential addition of all reagents, i.e., medium, fibrinogen, sodium hydroxide, acidic tropocollagen solution and finally thrombin. The mixture is performed in cold conditions (4 °C) and gel formation is triggered by incubation at 37 °C [121]. Slight modifications of the order of addition include introduction of thrombin in the cell culture medium (1′) [122,123,124,125] or preparation of a sodium hydroxide/medium/thrombin mixture to which acidic collagen and fibrinogen are added to obtain microbeads (1″) [126,127].

In other protocols, a defined mixture of two reagents is first prepared before addition to the others. For instance, acidic tropocollagen and fibrinogen are first mixed and then added to a NaOH-medium-thrombin solution (2) [128,129] or the tropocollagen/fibrinogen mixture is first neutralized before addition of the other components (3) [130,131,132,133,134,135]. A second possibility is to prepare a thrombin/fibrinogen mixture that then reacts with collagen (4) [136,137,138,139]. Finally, tropocollagen as well as atelocollagen are neutralized and mixed with thrombin before fibrinogen is added (5) [140,141]. Noticeably, an original protocol was recently described to obtain mixed threads by extrusion. In this case (6), acidic solutions of collagen, fibrinogen and thrombin are mixed, before being extruded in a buffer at neutral pH supplemented with polyethylene glycol (PEG) [111].

Type I tropocollagen is usually from bovine origin or extracted from rat tails tendons although goat tendon collagen has also been used [132]. Fibrinogen is from bovine or human sources but platelet-poor plasma [130] or commercial formulation (Tissuecol [138], Beriplast [141]) have also been used. In most cases, a defined concentration of CaCl_2_ is added to promote fibrin gel formation. Inhibitors of plasmin, such as aminocaproic acid (ACA), are also often present [121,124,142]. The use of glyoxal to cross-link the network was also reported [127]. Incorporation of hyaluronic acid [126,138] or elastin [128] was also described. Typical protein concentrations used in these protocols range between 1 and 5 mg mL^−1^. In most cases, 1:1 collagen:fibrin mixed hydrogels are prepared but several studies have explored a series of materials of intermediate concentrations [121,122,124,134,138,140,141,143].

To finish, an intermediate approach between the two previously-described ones was reported that highlights the diversity of protocols by which such mixed gels can be prepared. In this approach (7), collagen and fibrinogen were first mixed and incubated at neutral pH to achieved collagen gel formation. Then this gel is put in contact with thrombin to induce fibrin gel formation [144]. A similar but more complex approach combine collagen, alginate and fibrinogen. In this case, after collagen gel formation, calcium ions are added to form the alginate network and then thrombin is mixed to induce fibrin gelation [145].

## 4. Type I Collagen-Fibrin Mixed Hydrogels: Structure and Mechanical Properties

From a structural point of view, type I collagen and fibrin networks have strong similarity and, although fibrin fibrils tend to be thinner than collagen ones at similar concentration, it is usually difficult to unambiguously distinguish one protein from the other in SEM images [124,130,142]. TEM is more adapted to study fibril structure but, surprisingly, it was very rarely used to characterize mixed collagen-fibrin materials [111,145]. Confocal imaging using fluorescently-labelled proteins or, in the case of collagen, second harmonic generation microscopy, can allow to visualize the two networks but at a higher scale [130,143]. Thus, in many cases, the structure of the mixed networks is deduced from their mechanical behavior.

Gels obtained with protocol (3) (preliminary tropocollogen-fibrinogen mixture) were initially prepared at a 2:1 and 1:1 fibrin:collagen weight ratio and their behavior under compression was studied [130]. Initial elastic modulus and loss modulus were significantly larger in mixed gels compared to individual protein materials. All gels behave similarly as a function of compressive strain, i.e., an initial strain-softening at low strain, a constant response at intermediate strain and a strong stiffening at high strain. Decreasing protein concentration extended the plateau domain, i.e., softening and stiffening occurred at much lower, and much higher, respectively, strain values. SEM imaging showed thicker fibers and larger fiber bundles in compressed fibrin gels compared to collagen. However, the composite network showed intermediate structural features and the two proteins were not easily distinguished by this technique. Using confocal imaging, it was possible to study the connectivity of the networks. Density of nodes was larger in mixed gels than in collagen gels, with lowest value being obtained for fibrin gels. Under compression, the node density was increased for all materials. A more detailed analysis confirmed that the degree of connectivity between fibers was larger in the composites, mainly due to criss-cross junctions and fiber bundling, and was increased upon compression. Taken together, these results suggest that, in these conditions, the two protein networks are self-assembling independently and that the two structures interact by local fiber-fiber interactions only. Noticeably, by varying a number of experimental parameters such as collagen and fibrin concentration as well as temperature, it was possible to use this protocol to prepare mixed hydrogels with stress-strain curves and Young modulus close to those of rat myocardium [135].

**Figure 5:**
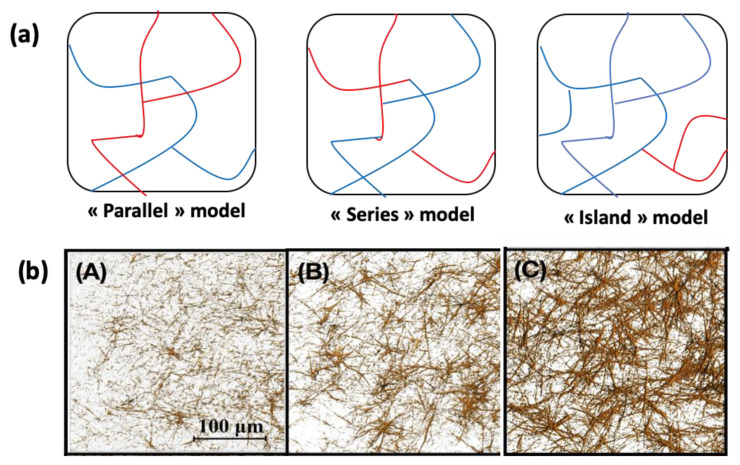
(**a**) Schematic representation of the possible structure of mixed type I collagen-fibrin gels; (**b**) second harmonic generation images of the collagen network in mixed gels with increasing collagen content from (**A**) to (**C**). In (**C**) collagen is not homogeneously dispersed in the network, in favor of the “Island” model. (**b**) is adapted and reproduced with permission from [143]. Copyright 2018 Acta Materialia Inc.

Gels obtained with protocol (4) (preliminary thrombin-fibrinogen mixtures) were prepared with various relative content in collagen and fibrinogen, and their strain-stress curves were both experimentally determined and calculated [136]. Experimentally, the Green strain at failure decreased and tangent modulus increased rapidly with increasing collagen concentration while the UTS remained almost constant. Two models were examined, a “parallel” one where two independent percolating networks are forming an interpenetrating structure and a “series” one where percolation occurs thanks to interconnexions between the two proteins forming a common network (Figure 5). It was found that the behavior of mixed gels could for some part be explained by the “series” model at low collagen content but was better reproduced by the “parallel” model at high collagen concentration. However, the fact that the UTS only slightly varied with gels composition could not be explained by these models. Moreover, further observations indicated that when one of the proteins is present in a small amount only, it does not form a percolating network. This raised the hypothesis of an “island” model, where the more diluted protein tends to locally concentrate rather than being homogeneously dispersed in the other protein network (Figure 5) [143]. Such a model was able to better reproduce experimental data in the low collagen and low fibrin composition domains. To understand better the mixed structures at comparable concentration, 1:1 collagen:fibrin hydrogels were prepared and digested by either collagenase or plasmin [137]. SEM imaging suggested that in the mixed gels, collagen fibers were slightly thinner compared to pure collagen hydrogels while fibrin fibers appeared more interconnected than in the fibrin-only structures. In terms of mechanical properties, no significant difference was found between the UTS value of single protein gels and mixed gels digested by their respective enzyme. However, Green Strain at failure was always smaller in digested gels, supporting the hypothesis that both collagen and fibrin networks present more connectivities within the mixed gels than when formed independently.

**Figure 6:**
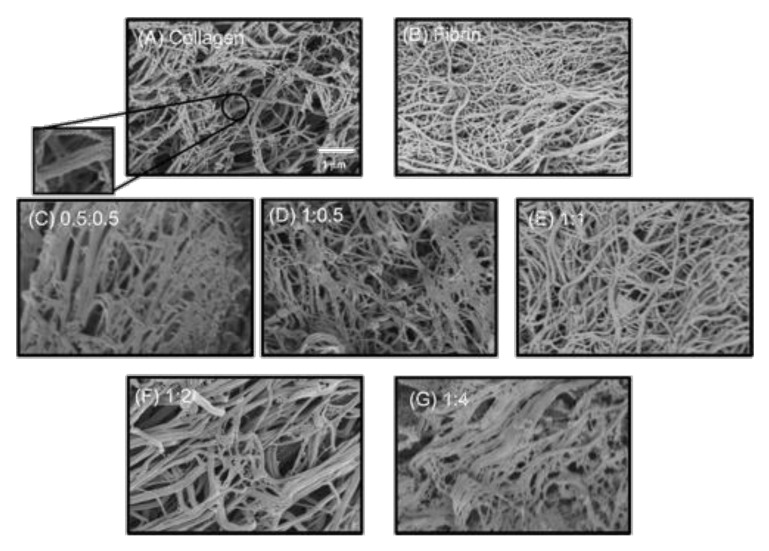
SEM images of pure collagen, pure fibrin and mixed hydrogels at various collagen:fibrin ratio prepared by protocol (1′). Reprinted with permission from [124]. Copyright 2006 American Chemical Society.

A series of studies was performed for hydrogels prepared by protocol (1) and (1′) in the presence of primary rat aortic smooth muscle cells (RASMC). Most characterizations were performed after 6 or 7 days of cell culture. In these conditions, gel compaction due to cell activity occurred, such a contraction being the least significant for pure collagen gels and the most marked for 1:1 fibrin:collagen mixed gels at fixed initial total protein concentration [121]. A specific shaping of the hydrogel as a ring structure foreseeing application as vascular biomaterials was studied by uniaxial testing, showing that the mixed hydrogel has intermediate modulus but higher UTS and toughness compared to single-protein materials. It was noticed that the values of all these mechanical features were lower when the initial protein concentration was increased but this has to be correlated with the accompanying lower extent of gel compaction. A more detailed analysis using various protein ratios (Figure 6) showed that final protein density was the highest when the total initial protein concentration was low (at fixed fibrin:collagen ratio) or the fibrin:collagen ratio was high (at fixed collagen concentration) [124]. Under uniaxial testing, all mixed gels showed higher modulus than single protein gels while their UTS values and toughness were much larger than the one of collagen and comparable or smaller than the one of fibrin. From the biological side, it was found that the number of cells was very similar in all constructs while, because of gel contraction, the constructs presenting either low initial protein content or low fibrin:collagen ratio exhibited the highest cell density. While these results are of interest for practical purposes, the fact that most mixed materials are prepared by varying both total protein content and protein ratio at the same time, and in concentrations different from the single protein materials, does not allow to draw conclusions about the collagen/fibrin interactions within these structures. Noticeably, further studies were performed to evaluate the influence of the enzyme that converts fibrinogen into fibrin on the mechanical properties of mixed gels [123]. Unfortunately, no data are provided on the fibrin-only hydrogels obtained in similar conditions. Moreover, it must be pointed out that muscle cells usually have a higher affinity for fibrin than for collagen which should contribute to some of the trends observed in these works.

Overall, in conditions where the two proteins are present in similar amounts, mixed hydrogels appear to be interpenetrating polymer networks (IPN) in which fibrin and collagen networks grow independently but can locally establish physical or structural interactions. This establishes additional cross-linking nodes that can increase the modulus of the mixed hydrogels compared to the single protein ones. Experiments performed in the presence of cells evidenced their ability to modify the mechanical properties of the mixed networks although the underlying structural changes were not investigated. This suggests that such mixed constructs can act as bifunctional 3D scaffolds for cell immobilization and tissue engineering applications, as described below.

## 5. Type I Collagen-Fibrin Mixed Hydrogels: Biomedical Applications

Collagen-fibrin mixed hydrogels obtained from protein mixtures have been prepared for several biomedical applications, mostly as cellularized materials (Table 1).

**Figure 7:**
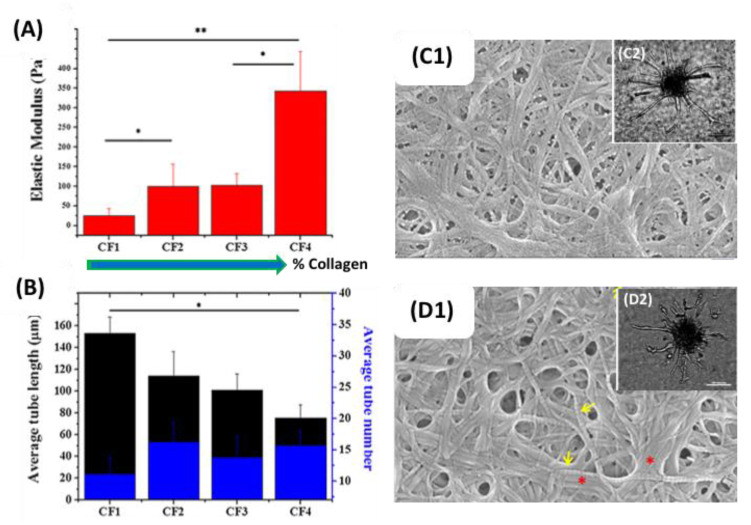
Angiogenesis by HUVECs cultured in Type I collagen-fibrin mixed hydrogels. Variation of (**A**) elastic modulus and (**B**) average tube length and tube number (collagen:fibrin weight ratio: CF1 1:4; CF2 2:3; CF3 3:2; CF4 4:1). SEM and optical images (as insets) for (**C**) CF2 and (**D**) CF3. ** *p* < 0.01, * *p* < 0.05. Yellow arrows indicate banded collagen fibrils. Red asterisks indicate bundles of fibrils. Adapted and reproduced with permission from [132]. Copyright 2018 Elsevier B.V.

A particular emphasis has been made in cardiovascular applications. For example, vascular smooth muscle cells (VSMCs) were encapsulated in pure collagen, pure fibrin and 1:1 collagen:fibrin constructs [142]. Expression of Collagen III was significant only in pure fibrin, collagen I expression was significant in mixed hydrogels, and to a lesser extent in pure collagen, and tropoelastin was expressed in the three matrices but in the order collagen > mixed > fibrin. The first one was mainly expressed one day after encapsulation while the expression of the two others increased with culture time. This was related to the physiological steps of wound healing where initial fibrin clots are first remodeled by deposition of type III collagen before elastin-incorporating type I collagen is formed. In that sense, the mixed hydrogels represent an artificial intermediary environment where only end products, i.e., fibrin and type I collagen, are present. In the same study, expression of integrin *β*3, *α*1 and *β*1 was also monitored. The former, a fibrin-binding integrin, was more expressed in pure fibrin while the two later, that are collagen-binding integrins, were prevailing in pure collagen. In all cases, the mixed hydrogels showed intermediate values between the two single proteins matrices. This clearly points out that cells are able to sense each protein independently and produce the adequate integrins to bind to both networks. Endothelial cells were also encapsulated to study angiogenesis. Human umbilical vein endothelial cell (HUVEC) spheroids were encapsulated in goat type I collagen:human fibrin mixed gels with weight ratio ranging from 1:4 to 4:1 [132]. The average tube length decreased with increasing ratio whereas the number of tubes was optimal for intermediate ratios, which was correlated with an increase in elastic modulus with collagen content. A higher number of branching points and tube connections was observed for the 2:3 composition compared to the 3:2, as attributed both to structural variations as well as an intrinsic higher ability of fibrin to promote sprouting compared to collagen (Figure 7).

**Figure 8:**
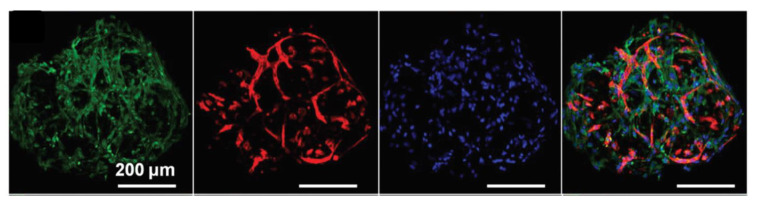
Co-culture of NHLFs (green) and endothelial cells (red) are co-localized (blue staining of nuclei) within collagen-fibrin microbeads. Reproduced with permission from [127]. Copyright The Royal Society of Chemistry 2014.

However, the response of endothelial cells to mixed hydrogels can also vary with their specific origin. Capillary formation by blood endothelial cells (BECs) on the one hand and by lymphatic endothelial cells (LECs) on the other, grown under interstitial fluid flow in vascular endothelial growth factor (VEGF)-containing mixed hydrogels, was compared [125]. BECs showed higher organization for highest relative collagen content while pure fibrin gels were the most favorable to LECs development. At a fixed collagen:fibrin ratio of 1:2, broad lumens were observed for BECs while fine ones were observed for LECs. In parallel, it was noticed a high rate of secretion of MMP-9 for LECs in collagen-rich matrices and for BECs in fibrin-rich ones, suggesting that both cells are trying to remodel the less favorable matrices. Proposed explanations include both mechanical and integrin-related cues as well as variations in matrix permeability, a key parameter in interstitial fluid culture conditions. However, the lack of experiments with a collagen-only hydrogel prevents to fully conclude on the contributions of each of these parameters. Co-culture of endothelial cells with other cell types has also been reported. For example, human mesenchymal stem cells (hMSCs) and HUVECs were co-immobilized in mixed hydrogels with various collagen:fibrin relative content [109]. At a fixed cell ratio, vasculogenesis, as monitored by numbers and connections of vessel-like structures, increased with the amount of fibrin, being the highest in the pure fibrin gel. A negative correlation was found between the storage modulus of the hydrogel and the vascular network extension. At a fixed collagen:fibrin ratio, reticulation by glyoxal led to an increase in modulus, negatively impacting on vasculogenesis. More recently, co-culture of normal human lung fibroblasts (NHLFs) and HUVECs in collagen-fibrin microbeads also led to efficient vasculogenesis (Figure 8) [127]. When such beads were entrapped within fibrin networks, angiogenetic processes could also be triggered, suggesting that these beads could be used for pre-vascularization of tissue engineering constructs.

These examples, as several others [134,135], illustrate the benefits of mixing collagen and fibrin but also point out that several parameters have to be considered to explain these benefits. From a structural perspective, the presence of fibrin usually decreases the elastic or Young modulus of collagen hydrogels. However, it enhances the porosity of the constructs, which is particularly favorable for vascularization. From a biochemical point of view, because the nature of integrins involved in the cell adhesion to the two proteins are different, it offers the possibility to co-culture two cell types with diverging affinities for type I collagen and fibrin. For instance, targeting tendon repair, tenoblasts and myoblasts have been grown on single-protein and mixed threads [111]. The former grow faster on collagen while the later efficiently colonize fibrin networks only. When cultured on mixed threads, tenoblasts showed intermediate behavior between the ones on single-protein hydrogels whereas myoblasts could colonize them to a comparable level to fibrin-only threads.

Despite the increasing amount of in vitro evidence of the benefits of combining type I collagen and fibrin within mixed hydrogels, there have been so far very few pre-clinical evaluations of their performances and, to our knowledge, no clinical studies. In many instances, these in vivo experiments in animal models are performed using only one hydrogel composition, that has been identified as the optimal one on the basis of preliminary in vitro evaluations. Thus, although they demonstrate the functionality of the selected biomaterial, these studies generally cannot demonstrate the benefits of mixed hydrogels over single-protein ones. For example, in the field of cardiovascular repair, mixed constructs with a collagen:fibrin weight ratio of 2:3 and containing HUVECs was shown to induce lumen formation after 7 days when implanted subcutaneously in mice, but no other compositions nor cell-free constructs were studied for comparison [132]. Accordingly, two studies showed the ability of collagen-fibrin mixed hydrogels seeded with chondrocytes to promote cartilage repair in rabbit or chicken [133,138]. However, these works focused on the benefits of the presence of the cells rather than on the composition of the host. Collagen-fibrin hydrogels containing DPSCs were also evaluated for dentin repair [129]. These hydrogels were prepared without or with bioglass particles and inserted in a tooth slice that was implanted in mice. After 8 weeks, the presence of the bioglass was shown to promote DPSCs differentiation into odonblast-like cells but the possible role of the collagen:fibrin ratio on this process was not investigated. On the contrary, collagen and collagen-fibrin (10%) hydrogels associated with Schwann cells were compared for nervous repair after transection of the sciatic nerve in rat models [134]. These experiments showed a higher number of axons within the construct containing fibrin after 4 weeks, which was attributed to its beneficial effect on the structure, the stability and the mechanical properties of the collagen network at this specific concentration.

## 6. Overview and Perspectives

Type I collagen and fibrin are two proteins that have a huge importance in tissue regeneration and have therefore been widely used to design biomaterials. From a physico-chemical point of view, they share the ability to self-assemble and form fibrillar hydrogels. However, they differ from a biochemical perspective, both in terms of structure and fibrillogenesis pathways. Therefore, although different protocols have been disclosed that allow for the formation of mixed hydrogels from protein solutions, evidences have been gathered that the resulting structures consist of two independent networks with local connections only. This should not come as a surprise due to the specificity of the existing interactions between monomers of a given protein, which makes the formation of mixed fibrils very unlikely. Achieving mixed fibers, i.e., co-assembly of collagen and fibrin fibrils, may not be out of reach. However, it will probably require to use a mixed colloid approach starting from pre-formed fibrils.

Indeed, Type I collagen and fibrin display distinct physiological roles and therefore induce different cell response. This probably constitutes the most significant interest of mixed constructs as they can allow the co-culture of two populations that would not efficiently colonize a common single-protein hydrogel. One promising perspective is to introduce other components, being structural or functional proteins or polysaccharides as well as mineral particles, to fit further with the specificity of the targeted tissue or pathology. Another possible development in this direction is to move from homogeneous co-gels to mixed materials exhibiting compositional, and therefore structural, gradients to control the orientation of cell growth.

## Figures and Tables

**Table 1:** Main biomedical applications of mixed type I collagen-fibrin hydrogels.

Targeted Tissue/Organ	Cells ^1^	Protocol ^2^	Reference
Bone	DPSCs	(2)	[129]
	hMSCs & HUVECs	(4)	[139]
Cardiovascular	RASMCs	(1), (1′)	[121,123,124]
	VSMCs	(1′)	[142]
	hMSCs & HUVECs	(1′)	[122]
	BECs & LECs	(1′)	[125]
	NHLFs & HUVECs	(1”)	[127]
	hMSCs & hASCs	(1”)	[126]
	HUVECs	(3)	[132]
	hiPSCs	(3)	[125]
Cartilage	Chondrocytes	(3), (4)	[133,138]
Lungs	- ^3^	(5)	[141]
Musculoskeletal	hMSCs	(7)	[145]
Nerve	Schwann cells	(3)	[134]
Pancreas	Pancreatic *β*-cells	(7)	[145]
Tendon	Satellite cells & tenoblasts	(6)	[111]

^1^ DPSCs: Dental Pulp Stem Cells; hMSCs: human Mesenchymal Stem Cells; HUVECs: Human Umbilical Vein Endothelial Cells; RASMCs: Rat Aortic Smooth Muscle Cells; VSMCs: Vascular Smooth Muscle Cells; BECs: Blood vascular Endothelial Cells; LECs: Lymphatic vascular Endothelial Cells; NHLFs: Normal Human Lung Fibroblasts; hASC: human Adipose Stem Cells; hiPSCs: human induced Pluripotent Stem cells; ^2^ see Figure 4; ^3^ acellular scaffolds.
